# HbA1c variability as an independent risk factor for diabetic retinopathy: results from a screening program

**DOI:** 10.1007/s00592-026-02711-7

**Published:** 2026-06-04

**Authors:** Maria Ida Maiorino, Carlo Gesualdo, Silvia Angelino, Settimio Rossi, Nicole Di Martino, Clemente Iodice, Miriam Longo, Lorenzo Scappaticcio, Giuseppe Bellastella, Francesca Simonelli, Katherine Esposito

**Affiliations:** 1 Department of Advanced Medical and Surgical Sciences, University of Campania “Luigi Vanvitelli”, Piazza Miraglia 2, 80138 Naples, Italy; 2 Division of Endocrinology and Metabolic Diseases, University Hospital, University of Campania “Luigi Vanvitelli”, Piazza Miraglia 2, 80138 Naples, Italy; 3 Eye Clinic, Multidisciplinary Department of Medical, Surgical and Dental Sciences, University of Campania, “Luigi Vanvitelli”, Naples, Italy; 4 Department of Life Sciences, Health and Health Professions, Link Campus University, Rome, Italy; 5 PhD Program of Translational Medicine, Department of Advanced Medical and Surgical Sciences, University of Campania “Luigi Vanvitelli”, Naples, Italy

**Keywords:** Diabetic retinopathy, Glucose variability, Type 1 diabetes, Type 2 diabetes, Retinopathy screening, HbA_1c_ coefficient of variation

## Abstract

**Aims:**

To investigate the independent association between long-term glycemic variability, measured as the coefficient of variation (CV) of HbA1c, and the presence of diabetic retinopathy (DR) in a cohort of adults with diabetes within a systematic eye screening program.

**Methods:**

This study screened 379 adults with type 1 and type 2 diabetes. Long-term glycemic variability was calculated as the CV of serial HbA_1c_ measurements. DR was assessed via dilated fundus photography. The association between HbA_1c_ CV and DR was analyzed using multivariable logistic regression adjusting for confounders.

**Results:**

DR incidence of 12.4%, mostly mild non-proliferative. The median HbA_1c_ CV was 7.0% (53 mmol/mol). In unadjusted bivariate analysis, the incidence of mild DR was higher in the low- glycemic variability group (CV < 5%) compared to the high- glycemic variability group. However, the multivariable logistic regression analysis revealed that higher HbA_1c_ CV was independently associated with the presence of DR (β = 0.643, P = 0.042), alongside diabetes duration (P < 0.001) and age (P < 0.001).

**Conclusions:**

In conclusion, in a cohort of individuals with both type 1 and type 2 diabetes and a low incidence of DR, long-term glycemic variability, estimated as HbA_1c_ CV, was independently associated with an increased risk of this complication.

**Supplementary Information:**

The online version contains supplementary material available at 10.1007/s00592-026-02711-7.

## Introduction

Diabetic retinopathy (DR) is a leading cause of blindness in adults and a major microvascular complication of diabetes [1]. Because DR often remains asymptomatic until advanced, vision-threatening stages, proactive surveillance is essential to avoid delayed diagnosis of complications such as macular edema or neovascularization. Since structured screening programs can prevent up to 98% of severe vision loss through early detection, they remain a cornerstone of modern diabetes management [1, 2].

The pathogenesis of DR is multifactorial, with prevalence and severity strongly correlated with diabetes duration [3]. Alongside established modifiable risk factors such as poor glycemic control, hypertension, and dyslipidemia, recent research has increasingly focused on the clinical impact of long-term glycemic variability, measured via serial HbA_1c_, as a potential predictor for the disease [4–6].

Glycemic variability is a heterogeneous cluster of glycemic dysregulations defined by either short- or long-term fluctuations of glucose levels [7–9]. The long-term glycemic variability quantifies glycemic fluctuations over extended periods, typically months or years. It is commonly assessed through visit-to-visit changes in glycemic markers, including serial measurements of HbA_1c_, fasting plasma glucose, and postprandial glucose. Metrics such as the standard deviation (SD) and the CV are frequently used to calculate this long-term variability [6, 10, 11].

It is now understood that people with diabetes may exhibit significant long-term glycemic variability even with similar HbA_1c_ values. This visit-to-visit variability has emerged as an important predictor for adverse outcomes in diabetes, including micro- and macro-vascular complications and mortality [6, 10–16]. Interestingly, recent systematic reviews and meta-analyses showed that HbA_1c_ variability is positively associated with the risk of developing microvascular complications and cardiovascular diseases, even independently of mean HbA1c [6, 16]. Furthermore, the impact of HbA_1c_ variability in individuals with type 2 diabetes on the development of vascular complications appears to be comparable to that of major established risk factors, including age, male sex, and a history of hypoglycemia. This suggests that HbA_1c_ variability may exert a greater influence on the disease progression than the HbA_1c_ levels themselves [16]. With regard to the predictive role of HbA_1c_ on the development of diabetic retinopathy, emerging evidence from observational studies suggests a link between glycemic variability and DR, with the association appearing more consistent in type 1 than in type 2 diabetes [6, 7, 12, 17–19], although its definitive role remains to be fully elucidated.

Therefore, taking advantage of a joint eye screening program at a Tertiary Diabetes Center, the present study aims to investigate the independent association between long-term glycemic variability—specifically measured as HbA_1c_ CV over a 5-year period—and the presence of DR in a real-world cohort of adults with type 1 and type 2 diabetes. This approach allows us to clarify the role of HbA_1c_ CV as a potential metric for refined risk stratification in clinical practice.

## Subjects, materials and methods

### Study design and population

This was a monocentric, retrospective study carried out at the tertiary Diabetes Center of the Unit of Endocrinology and Metabolic Diseases of University of Campania “Luigi Vanvitelli” (Naples, Italy). The project was developed within a joint DR screening program in collaboration with the University Hospital’s Ophthalmology Unit. The study protocol was conducted in conformity to the Strengthening the Reporting of Observational Studies in Epidemiology (STROBE) Statement [20], and the corresponding checklist is reported in the Online Only Supplemental Material.

We consecutively enrolled all adults with a confirmed diagnosis of type 1 or type 2 diabetes from June 2023 to June 2025 who have a minimum continuous follow-up of at least 5 years at the Diabetes Center and did not met the following exclusion criteria: 1) diabetes diagnosis not clear nor confirmed, 2) acute or chronic conditions (assessed with an accurate anamnesis and laboratory tests) that could influence glucose variability, 3) incomplete clinical records, 4) follow-up discontinuity during the observation period; 5) fewer than 10 HbA_1c_ measurements over the preceding five years, or 6) a previous diagnosis of DR. All participants underwent an ophthalmological evaluation that included dilated fundus examination and color fundus photography, and HbA_1c_ measurements to calculate the variability and retrospectively collected from the medical records of the previous 5-years follow-up, as summarized in Supplementary Figure S1. The study was approved by the Medical Ethics Committee of University of Campania “Luigi Vanvitelli” (Prot. 0003239/I, 01/02/2023) and complies with the Declaration of Helsinki and the International Conference on Harmonization/Good Clinical Practice Guidelines. All participants signed informed consent before the enrollment.

### Clinical variables

All clinical and laboratory data were collected for each participant during the recruitment visit, which coincided with the ophthalmological screening. This baseline assessment included age, sex, type and duration of diabetes, current medication, and smoking status. Anthropometric measurements, including body weight and height, were used to calculate the body mass index (BMI). Overweight and obesity were defined according to the World Health Organization (WHO) criteria [21]. Blood pressure was measured according to standard procedures, and hypertension was defined as an average blood pressure ≥ 140/90 mmHg or the current use of antihypertensive medication. Fasting plasma glucose, HbA_1c_, and full lipid profile (total, HDL, and LDL cholesterol, and triglycerides) were collected from medical records exhibited by patients. Hyperlipidemia was diagnosed in the presence of LDL cholesterol > 160 mg/dL, HDL cholesterol < 40 mg/dL for men or < 50 mg/dL for women, or triglycerides > 150 mg/dL, or current use of lipid-lowering therapy. Data regarding the presence of atherosclerotic disease and other diabetes-related micro and macrovascular complications were also recorded.

### Assessment of glycemic variability

Long-term glycemic variability was assessed using the CV of serial HbA_1c_ measurements, a well-established metric for visit-to-visit fluctuations. [11]. For each participant, two HbA_1c_ measurements per year from the five-year follow-up period preceding the DR assessment were retrospectively collected from their electronic medical records. These values were compiled into a dedicated internal database by study physicians. The CV was calculated for each patient using the following formula:

$${\text{CV (\% ) = (SD/mean HbA}}_{{{\mathrm{1c}}}} {\mathrm{)}} \times {\mathrm{100,}}$$

where SD corresponds to standard deviation [9]. A CV value equal or superior to 5% was used as the threshold to identify the participants as having high long-term glycemic variability, a cut-off supported by evidence linking it to adverse clinical outcomes [12].

### DR assessment

For each enrolled patient, dilated fundus examination was performed, and color fundus photographs were acquired in both eyes using a digital non‐mydriatic camera (CenterVue® DRS Automatic Retinal Camera) during the one-time recruitment visit. DR was retrospectively graded by two independent experienced ophthalmologist (C.G. and C.I.) basing on the International Clinical Diabetic Retinopathy Disease Severity Scale [22] as follows: no apparent retinopathy; mild non-proliferative DR (NPDR), featuring microaneurysms only; moderate NPDR, with microaneurysms, dot and blot hemorrhages, hard exudates, or cotton wool spots but less than severe NPDR; severe NPDR, characterized by minimum 20 intraretinal hemorrhages in each of the 4 quadrants, venous beading in 2 quadrants, or intraretinal microvascular abnormalities (IRMA) in 1 quadrant; and proliferative DR (PDR), featured by findings of neovascularization.

### Statistical analysis

The primary objective of this study was to investigate the independent association between long-term glycemic variability —calculated as the HbA_1c_ CV over the five-year follow-up period—and the presence of diabetic retinopathy. As a secondary outcome, we assessed the incidence of newly diagnosed DR within the cohort of patients through the systematic eye screening program. The sample size was originally calculated to estimate the prevalence of diabetic retinopathy in our population with sufficient precision. Based on an expected prevalence of 22% [2], a minimum sample size of 264 participants was required to achieve a 95% confidence interval with a 5% margin of error. The final study cohort included 379 participants, exceeding this minimum requirement. This sample size provided sufficient statistical power to perform the multivariable logistic regression analysis, maintaining an adequate ratio of events (new DR diagnoses) per predictor variable, in line with current recommendations for robust clinical association studies.

Descriptive statistics were used to summarize the baseline characteristics of the study population. The Kolmogorov–Smirnov test was used to analyze the distribution of the continuous variables. Data were presented as median and interquartile ranges (IQ) for continuous variables and as frequencies and percentages for categorical variables. The cohort was then stratified based on HbA_1c_ CV into two groups: high variability (CV ≥ 5%) and low variability (CV < 5%). Differences between groups were analyzed using the Mann–Whitney rank sum test for continuous variables and the Chi-Square test for categorical variables, respectively. A binary logistic regression model was employed to evaluate the association between the independent variable (HbA_1c_ CV) and the dependent variable (incidence of diabetic retinopathy). To control for confounding, the model was adjusted for covariates known to be associated with both dependent and independent variables, including diabetes duration, age, hypertension status, current smoking habit, LDL cholesterol, total cholesterol, and BMI). The predicted probabilities were calculated using the standard logistic regression formula:

$${\text{P(Y = 1|X) = e}}\left( {\beta 0 + \beta 1X} \right) + e\left( {\beta 0 + \beta 1X} \right)$$

## Results

### Study cohort characteristics

A total of 574 patients attending the Diabetes Center received the fundus examination in the eye screening program for diabetic retinopathy. Of these, 195 participants were excluded from the final analysis because they lacked a sufficient number of serial HbA_1c_ measurements to calculate glycemic variability; therefore, the final study cohort, comprised 379 participants. The demographic and clinical characteristics of the study population are summarized in Table 1. The study population was equally distributed for sex, with 165 females (43.5%) and 214 males (46.5%), and the median age was 60.0 years old. The majority of patients had type 2 diabetes (60.7%), with a median diabetes duration of 12.0 years. The median BMI resulted 27.4 kg/m^2^, with 36.4% of the study population being overweight/obese. HbA_1c_ was 7.0% (53 mmol/mol), and the median HbA_1c_ CV was also 7.0%. The overall prevalence of microvascular complications was 19.0%, with diabetic neuropathy being the most common (10.0%), whereas macrovascular complications were present in 70 subjects (18.5%) (Supplemental Figure S2). Regarding diabetes treatment, among participants with type 1 diabetes the most common regimen (47%) was continuous subcutaneous insulin infusion (CSII) combined with continuous glucose monitoring (CGM), followed by multiple daily insulin injections (MDI) and CGM (36.7%) (Supplemental Figure S3). In participants with type 2 diabetes, metformin monotherapy was the most frequent treatment (35.4%), followed by metformin plus injective drugs (27.6%), and metformin plus oral drugs (16.8%) (Supplemental Figure S4).

**Table 1:** Variables of interest in the entire study cohort

Variables	Entire study cohort (n = 379)
Age, years	60.0 (40.0–70.0)
Type 1 Diabetes, n (%)	147 (38.8)
Type 2 Diabetes, n (%)	230 (60.7)
Other types of diabetes, n (%)	2 (0.7)
Diabetes duration, years	12.0 (5.0–20.0)
Female, n (%)	165 (43.5)
Current smokers, n (%)	100 (26.4)
Body weight, kg	77.0 (67.0–87.0)
BMI, kg/m^2^	27.4 (24.1–31.1)
Overweight/obesity, n (%)	138 (36.4)
SBP, mmHg	125.0 (120.0–130.0)
DBP, mmHg	75.0 (70.0–80.0)
Fasting plasma glucose, mg/dL	128.0 (108.0–151.0)
HbA_1c_, %	7.0 (6.4–7.8)
HbA_1c_, mmol/mol	53 (46–62)
HbA_1c_ CV, %	7.0 (4.6–10.4)
Total Cholesterol, mg/dL	158.0 (135.0–185.0)
HDL Cholesterol, mg/dL	51.0 (42.3–61.0)
LDL Cholesterol, mg/dL	84.6 (63.2–107.5)
Triglycerides, mg/dL	89.0 (62.3–134.0)
Hypertension, n (%)	159 (42.0)
Atherosclerotic disease, n (%)	90 (23.7)
Hyperlipidemia, n (%)	190 (50.1)
New diagnosis of Diabetic Retinopathy, n (%)	47 (12.4)
Mild Proliferative Diabetic Retinopathy, n (%)	34 (72.3)
Moderate Proliferative Diabetic Retinopathy, n (%)	10 (21.2)
Severe Proliferative Diabetic Retinopathy, n (%)	1 (2.3)
Proliferative Diabetic Retinopathy, n (%)	2 (4.2)

*BMI* body mass index, *DBP*, diastolic blood pressure, *HDL* high density lipoprotein, *LDL* low density lipoprotein, *SBP* systolic blood pressure

### Incidence and characteristics of DR

**Fig. 1:**
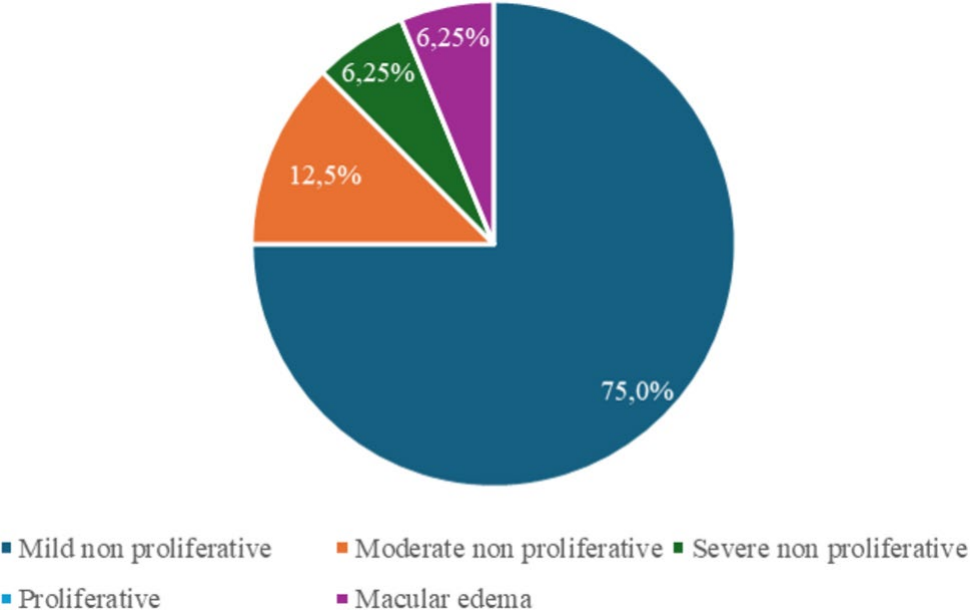
Distribution of diabetic retinopathy stages identified by the screening program

The screening program identified a new diagnosis of diabetic retinopathy (DR) in 47 participants (12.4% of the total cohort). Among these newly diagnosed cases, the majority presented with mild non-proliferative DR (NPDR) (72.3%), followed by moderate NPDR (21.2%). Severe NPDR and proliferative DR were less frequent (2.3% and 4.2%, respectively) (Table 1, Fig. 1). The incidence of new DR diagnosis according to the HbA_1c_ CV was described in Table 2. The 273 (72%) participants with high glycemic variability (HbA_1c_ CV ≥ 5%) had a significantly longer diabetes duration [median (IQ), 13.0 (6.8–20.0) vs 9.0 (4.0–15.0) years, P = 0.002] and lower total cholesterol levels [155.0 (133.0–181.0) vs 167.0 (143.0–196.0), P = 0.040] compared to the 106 subjects with low glycemic variability (HbA_1c_ CV < 5%). The two groups were similar for the other explored clinical parameters. Although the overall incidence of newly diagnosed DR did not differ significantly between the high- glycemic variability and low- glycemic variability groups (10.6% vs. 17.0%, P = 0.131), the incidence of mild NPDR was significantly lower in the high-glycemic variability group (7.0% vs. 14.2%, P = 0.046).

**Table 2:** Variables of interest in the study cohort stratified according to HbA_1c_ CV

Variables	CV HbA_1c_ ≥ 5% (n = 273)	CV HbA_1c_ < 5% (n = 106)	P
New DR, n (%)	29 (10.6)	18 (17.0)	0.131
Mild, n (%)	19 (7.0)	15 (14.2)	0.046
Moderate, n (%)	7 (2.6)	3 (2.8)	0.832
Severe, n (%)	1 (0.4)	0 (0)	0.623
Proliferative, n (%)	2 (0.7)	0 (0)	0.925
Anamnestic DR, n (%)	11 (4.0)	5 (4.7)	0.989
Age, years	58.0 (33.0–69.0)	59.5 (43.0–70.0)	0.236
Diabetes duration, years	13.0 (6.8–20.0)	9.0 (4.0–15.0)	0.002
Female, n (%)	126 (46.2)	38 (35.8)	0.089
Current smokers, n (%)	71 (26.0)	29 (27.4)	0.890
Body weight, kg	75.3 (66.0–85.0)	78.0 (68.0–87.0)	0.196
BMI, kg/m^2^	27.1 (23.8–31.0)	27.4 (23.7–31.2)	0.862
Overweight/obesity, n (%)	96 (35.2)	42 (39.6)	0.490
Fasting plasma glucose, mg/dL	128.0 (105.0–151.0)	127.0 (109.0–150.0)	0.622
HbA_1c_, %	7.0 (6.4–7.6)	7.0 (6.4–7.7)	0.959
HbA_1c_, mmol/mol	53 (46–60)	53 (46–61)	0.959
HbA_1c_ CV, %	8.4 (7.1–12.5)	3.2 (2.1–4.2)	< 0.001
Total Cholesterol, mg/dL	155.0 (133.0–181.0)	167.0 (143.0–196.0)	0.040
HDL Cholesterol, mg/dL	51.0 (43.0–61.0)	53.0 (45.0–64.0)	0.254
LDL Cholesterol, mg/dL	82.8 (63.2–103.3)	88.5 (69.2–111.6)	0.058
Triglycerides, mg/dL	88.0 (60.0–134.0)	88.0 (63.0–132.0)	0.485
Hypertension, n (%)	112 (41.0)	46 (43.4)	0.761
Atherosclerosis, n (%)	62 (22.7)	28 (26.4)	0.531
Hypercholesterolemia, n (%)	137 (50.2)	53 (50.0)	0.934
Diabetic Neuropathy, n (%)	31 (11.4)	6 (5.7)	0.105
Diabetic Nephropathy, n (%)	5 (1.8)	3 (2.8)	0.834
Diabetic Foot, n (%)	10 (3.7)	0 (0)	0.101
Macrovascular Complications, n (%)	50 (18.3)	20 (18.9)	0.982

*BMI* body mass index, *CV* coefficient of variation, *DR* diabetic retinopathy, *HDL* high density lipoprotein, *LDL* low density lipoprotein

### Association between HbA_1c_ CV and diabetic retinopathy

In the binary logistic regression analysis, after adjusting for potential confounders, a higher HbA_1c_ CV was independently associated with an increasing likelihood of having DR (β coefficient = 0.643, P = 0.042). Other factors significantly associated with DR were longer diabetes duration (β coefficient = 0.051, P < 0.001), older age (β coefficient = 0.041, P < 0.001), and serum lipid levels (LDL and the total cholesterol) (Table 3).

**Table 3:** Multiple logistic regression analysis between the dependent variable (presence of diabetic retinopathy) and the HbA_1c_ CV, after adjustment for diabetes duration, age, hypertension, smoking status, LDL cholesterol, total cholesterol, BMI

Variables	β coefficient	P
CV HbA_1c_	0.643	0.042
Diabetes duration, years	0.051	< 0.001
Age, years	0.041	< 0.001
Hypertension, yes/no	0.076	0.816
Current smoking status, yes/no	0.223	0.510
LDL cholesterol, mg/dl	0.026	0.033
Total cholesterol, mg/dl	−0.023	0.024
BMI, kg/m^2^	0.025	0.370

*BMI* body mass index, *CV* coefficient of variation, *LDL* low density lipoprotein

## Discussion

To the best of our knowledge, this is the first study to investigate the incidence of DR within the framework of a joint screening initiative at a Tertiary Diabetes Center in Southern Italy. A unique aspect of our work was the simultaneous investigation of the association with long-term glucose variability, calculated over a minimum follow-up of 5 years, and the incidence of DR in this cohort.

Our study focused on a group of adults with DM, with the majority having type 2 diabetes, and characterized by a fair glyco-metabolic control. The study population reported a low incidence of diabetes-related microvascular complications, with diabetic neuropathy being the most representative. Our screening program revealed a DR incidence of 12.4%, with most cases classified as mild NPDR. This rate is considerably lower than the global prevalence of approximately 22% and the Italian published prevalence, which ranges from 15.5 to 27.6% [2, 23]. This finding characterizes the clinical context of our cohort, since we hypothesized that this difference reflects the efficacy of the comprehensive and specialized care provided at our tertiary-level diabetes center. This interpretation is supported by the cohort’s overall fair glyco-metabolic profile, including well-managed dyslipidemia despite a high prevalence of hypercholesterolemia. The observed low incidence of DR is therefore consistent with the well-established evidence that structured, long-term follow-up and intensive glycemic control significantly reduce the risk of microvascular complications [24–27].

Our assessment of long-term glycemic variability was based on serial HbA_1c_ measurements collected two times a year over five years. We observed that the median HbA_1c_ CV in our cohort exceeded the proposed target of 5% despite fair overall glycemic control as indicated by the median HbA_1c_ level [12]. A potential explanation for this elevated glycemic variability is that our observation period partially overlapped with the COVID-19 pandemic. It is well documented that public health restrictions during this time led to significant disruptions in routine diabetes care, including discontinuation of face-to-face visits, which may have impacted glycemic stability [28].

A key finding is that after adjusting for traditional risk factors, higher long-term glycemic variability, measured by HbA_1c_ CV, was independently associated with the presence of DR in our cohort. This result is particularly noteworthy when considering our initial bivariate analysis, which revealed a counterintuitive higher incidence of new DR cases (specifically mild NPDR) in the low- glycemic variability group (CV < 5%). This apparent paradox is underscored by the fact that the low- glycemic variability group also had a significantly shorter diabetes duration, a factor that is typically protective against the development of retinopathy. This discrepancy strongly suggests that the unadjusted analysis is influenced by significant confounding. Therefore, the multivariable logistic regression model—by adjusting for duration, age, and other covariates—was essential to isolate the true, independent detrimental effect of high HbA_1c_ CV [15, 19, 29].

The association between high glycemic variability and DR is supported by several proposed pathogenetic mechanisms, as summarized by Kilpatrick [29]. First, high glycemic variability may act through a mechanism analogous to the “metabolic memory”, whereby transient but repeated hyperglycemic episodes induce persistent vascular stress and epigenetic changes [30, 31]. Second, because the risk of microvascular complication rises exponentially with increasing HbA_1c_, individuals with high variability of HbA_1c_ spend more time at the upper, more damaging end of their HbA_1c_ range, which disproportionately elevates their overall risk [32]. Third, the phenomenon of “early worsening” of DR, after rapid improvement in glycemic control, suggests that large glucose fluctuations themselves can be detrimental to the retinal vasculature [33].

Nevertheless, we acknowledge that the role of long-term glycemic variability as an independent risk factor for DR remains a subject of debate. The findings in literature are inconsistent, with several studies suggesting the role of HbA_1c_ CV as an independent risk factor for the occurrence of DR [15, 19, 29]. On the other hand, there is evidence from observational studies that the association between visit-to-visit HbA_1c_ variability and DR is attenuated or becomes non-significant after adjusting for mean HbA_1c_ levels, particularly in cohorts with type 2 diabetes [20, 34]. Our findings contribute to the growing body of evidence supporting the clinical relevance of monitoring long-term glycemic variability as a potentially modifiable risk factor. We suggest that integrating HbA_1c_ CV into routine care could provide clinicians with a refined tool for risk stratification, potentially guiding decisions on the frequency of ophthalmological follow-up and the intensification of glucose-lowering therapy. In our study, high variability was defined using a threshold of CV equal or higher than 5%, calculated over a 5-year data window with a minimum of 10 measurements. While these parameters proved effective for identifying patients at higher risk of DR in our cohort, we acknowledge that it is currently not possible to establish a universal clinical protocol. Moreover, since the retrospective calculation of HbA_1c_ CV may be time-consuming in a typical follow-up visit, clinicians might prioritize this metric for a specifical subset of those patients experiencing oscillations between periods of glycemic stability and decompensation. Towards a clinical application, further evidence is required to standardize the optimal timing of assessment, define a validated ‘high-risk’ cutoff, and determine specific management changes—such as earlier referral or personalized screening intervals—to be implemented in routine clinical practice.

This study has many strengths, including 1) a well-sized cohort, with a similar proportion of female and male participants; 2) the inclusion of participants who had an history of at least five-years follow-up at our Diabetes Unit, that allowed us to assess the HbA_1c_ CV based on a considerable number of HbA_1c_ measurements; 3) the comprehensive collection of clinical and biochemical data, that allowed us to adjust the analyses for a wide range of potential confounders.

Our findings should be interpreted also considering several limitations. The primary limitation is the retrospective study design, which allows us to identify an association, but does not permit the inference of causality between high HbA_1c_ CV and DR. Furthermore, the design precludes a formal evaluation of the predictive value of glycemic variability for and the future progression of DR. Second, the biochemical data, including the HbA_1c_ measurements, were sourced from routine clinical records rather than a central laboratory. This introduces the possibility of inter-assay variability, although it also enhances the real-world applicability of our findings.

In conclusion, in a cohort of individuals with both type 1 and type 2 diabetes with a low incidence of DR, long-term glycemic variability, estimated as HbA_1c_ CV, is independently associated with an increased risk of this complication. Our findings come from an eye screening program for early DR detection and highlight the importance of integrating glycemic variability assessment into clinical practice for enhanced risk stratification. Further prospective studies are warranted to confirm the predictive role of long-term glycemic variability in the development and progression of the microvascular diabetes-related complications, particularly DR, also analyzing possible associations with recognized ocular biomarkers.

## Supplementary Information

Below is the link to the electronic supplementary material.

Supplementary file1 (DOCX 70 KB)

## Data Availability

All data generated or analyzed during this study are included in the published article (and its online supplementary files).
